# The Use of Laparoscopic Camera Sleeve in Implant-Based Breast Reconstruction: An Innovative “No-Touch” Technique

**DOI:** 10.1007/s00266-026-05720-7

**Published:** 2026-03-31

**Authors:** Mahmoud Ahmed Alhussini, Ali Mohamed Elameen, Ahmed Mohamed Abouzaid, Mohamed Mahmoud Soffar

**Affiliations:** 1 Surgical Oncology Unit, General Surgery Department, Faculty of Medicine, Alexandria University, 22 El-Geish Road, El-Shatby, Alexandria, Egypt; 2 Department of Plastic and Reconstructive Surgery, El-Sahel Teaching Hospital, Cairo, Egypt; 3 Plastic and Reconstructive Surgery Department, El-Gomhoria Hospital, Alexandria, Egypt; 4 Plastic and Reconstructive Surgery Unit, Department of General Surgery, Faculty of Medicine, Alexanderia University, Alexandria, Egypt

**Keywords:** No-Touch, Breast implants, Reconstruction, Laparoscopic camera sleeve

## Abstract

**Background:**

Implant-based breast reconstruction (IBBR) is the predominant reconstructive modality following mastectomy, accounting for more than 80% of all breast reconstruction procedures. Preventing subclinical infection at the time of implant insertion is critical for reducing the incidence of capsular contracture. This study evaluated a simple, cost-effective, flexible, and disposable laparoscopic camera sleeve designed to facilitate the no-touch technique in breast implant insertion.

**Methods:**

A prospective study was conducted between November 2021 and September 2025, including all patients undergoing IBBR with the laparoscopic camera sleeve technique. The narrow end of the sleeve was removed and employed as an additional cover for the implant. The prepared sleeve was partially introduced into the pocket, allowing the implant to be inserted without contact with the skin.

**Results:**

A total of 42 patients underwent IBBR using smooth round implants, with a mean implant size of 432.85 ± 69.68 ml. Postoperative seroma occurred in seven patients (16.7%), and wound dehiscence in two patients (4.8%). No implant rupture, postoperative infection, or capsular contracture was observed during the follow-up period. Ten patients (23.8%) received adjuvant radiation therapy, with a mean follow-up of 18.87 ± 8.86 months.

**Conclusion:**

The use of the laparoscopic camera sleeve provides a simple, cost-effective, and reproducible technique for no-touch insertion of breast implants. The technique substantially reduces skin contact and potential contamination, thereby lowering the risk of postoperative infection and capsular contracture.

**Level of Evidence III:**

This journal requires that authors assign a level of evidence to each article. For a full description of these Evidence-Based Medicine ratings, please refer to the Table of Contents or the online Instructions to Authors www.springer.com/00266.

**Supplementary Information:**

The online version contains supplementary material available at 10.1007/s00266-026-05720-7.

## Introduction

Aesthetic and reconstructive breast surgeries are among the most performed surgical procedures worldwide. In the USA, more than 500,000 patients are undergoing aesthetic breast surgeries annually, in which augmentation mammoplasty is the most common procedure [1, 2]. Implant-based breast reconstruction (IBBR) is the predominant reconstructive modality following mastectomy, accounting for more than 80% of all breast reconstruction procedures [3]. Notably, more than 35 million women are wearing breast implants globally, with more than 600,000 implants used yearly in the USA [4–6]. The widespread demand for aesthetically pleasing breast restoration, coupled with the desire for satisfactory functional and physical outcomes, continues to drive the global rise in implant-based breast surgeries [7]. However, implant infection is associated with poor wound healing, suboptimal cosmetic results, and reconstructive failure. Furthermore, long-term complications of breast implants mostly include capsular contracture, which causes chronic pain, disfigurement, and poor quality of life [8, 9]. Preventing subclinical infection at the time of implant insertion is critical for reducing the incidence of capsular contracture, which occurs in 5–74% of cases and represents a leading indication for revision surgery [10].

A wide variety of preoperative, intraoperative, and postoperative procedures have been adopted to reduce the risk of bacterial contamination during breast implant surgeries [11]. Biofilm-related breast implants are mostly caused by bacterial seeding from the skin during the insertion of the implant. The predominant component of implant contamination is from the bacteria of the nipple–areola complex (NAC) and surrounding skin. Despite adequate irrigation of the pocket and sterilization of the skin, positive skin cultures can still be obtained [12–14]. The relationship between bacterial contamination and capsular contracture has been established. This relationship led to the development of the “no-touch” technique to decrease skin contamination and biofilm formation. The technique aims to insert the implant into the pocket without contact with skin edges, which are potential sources of implant trauma and bacterial colonization. The use of the Keller Funnel considerably minimizes bacterial contamination, provides easier insertion of breast implants, and subsequently reduces the risk of capsular contracture [15]. The funnel makes breast implant insertion safe by reducing the shell trauma to the implant and the contact with the surgeon’s gloves during insertion and the patient’s skin [16]. However, the use of the Keller Funnel is costly, adding additional burden to the patients and facilities, particularly in low-income countries [17]. The modified Toomey syringe casing was used for easy insertion of breast implants; however, the technique requires cutting the casing with a saw and burning it [18]. The present study introduces a simple, cost-effective, flexible, and disposable laparoscopic camera sleeve for facilitating a no-touch insertion technique during breast implant reconstruction.

## Methods

The study was conducted in accordance with the ethical guidelines of the Ethics Unit, Faculty of Medicine, Alexandria University, Alexandria, Egypt (Registration number: 0306965). Potential benefits and risks associated with the laparoscopic camera sleeve technique were explained to all patients prior to surgery. The study adhered to the principles outlined in the Declaration of Helsinki [19]. The present study was performed following the strengthening reporting of observational studies in epidemiology (STROBE) statement guidelines for conducting prospective studies [20].

### Study Design

This prospective study was performed between November 2021 and September 2025. The study was conducted at the Surgical Oncology Department at Alexandria University Hospital, Faculty of Medicine, Alexandria University, Egypt.

### Eligibility Criteria

All patients with IBBR who were subjected to the laparoscopic camera sleeve technique were included. Patients lost to follow-up or patients with less than six months of postoperative follow-up were excluded.

### Surgical Technique

**Fig. 1:**
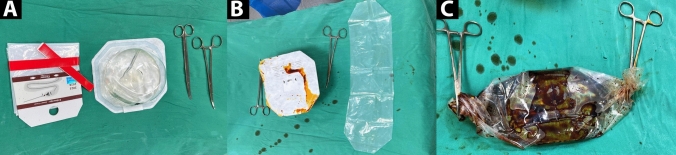
Preparation of the laparoscopic camera sleeve for “no-touch” implant insertion. (**A**) Original materials including the implant, laparoscopic camera sleeve, and surgical instruments used for preparation. (**B**) The sleeve is trimmed to an appropriate length with additional back cuts to facilitate implant passage and may be soaked with Betadine for lubrication and antisepsis. (**C**) The implant was placed into a laparoscopic camera sleeve pre-soaked in Betadine, allowing for insertion into the breast pocket without direct skin contact

The narrow end of the laparoscopic camera sleeve was cutoff, and the sleeve was used as an additional cover for the implant during its insertion into the pocket. The sleeve was prepared simply by cutting a reasonable length (around 30–40 cm). Additional back cuts were made to facilitate the insertion of the implant into the sleeve. The laparoscopic sleeve was placed through the inframammary incision toward the pocket, with implants inserted without skin contact. The procedure was applied either during insertion or prior to insertion. The prepared sleeve was partially introduced into the pocket, where one end was in the pocket and the other remained outside. The implant was squeezed into the sleeve and then directly squeezed into the pocket without coming into direct contact with the skin. The implant was squeezed into the prepared sleeve over a draped surgical table. A surgical instrument grasped both ends of the sleeve. One end was pushed into the pocket, and then the implant was squeezed into the wound, applying downward pressure to slide the implant into the pocket without requiring contact with the surgeon’s gloves or the surrounding skin. The surgeons advanced the implant into the pocket with minimal force and no finger manipulation. After insertion, the sleeve was gently pulled out to keep the implant in place with the other hand. The operating surgeon checked the sleeve after extraction to ensure it was intact. The surgeon avoided trials of implant squeezes from outside the sleeve, which carried the risk of tearing it. The authors preferred to soak the sleeve with Betadine on both the outside and inside. This acted as an additional lubricant, facilitating the passage of the implant through the sleeve and providing an extra measure to reduce the risk of infection (Fig. 1 and Supplementary Video).

### Data Collection and Study Endpoints

Data were collected in a well-structured Excel sheet. Patient demographics, including age, as well as breast cancer-related information, such as cancer stage and the number of affected breasts, were recorded. Variables associated with surgical procedures, including the pattern of mastectomy, type of breast-conserving surgery, timing of reconstruction, implant pocket location, implant size, and implant coverage, were extracted. The study endpoint was defined as the number of patients who developed capsular contracture by the end of the follow-up period.

### Statistical Analysis

Continuous variables with normal distribution were reported as mean ± standard deviation, whereas categorical variables were expressed as numbers and percentages. Statistical analyses were conducted using SPSS version 25 (SPSS Inc., Chicago, IL, USA) [21]. Figures were renovated using GraphPad Prism (GraphPad Software, Inc, San Diego) software version 8 [22].

## Results

**Fig. 2:**
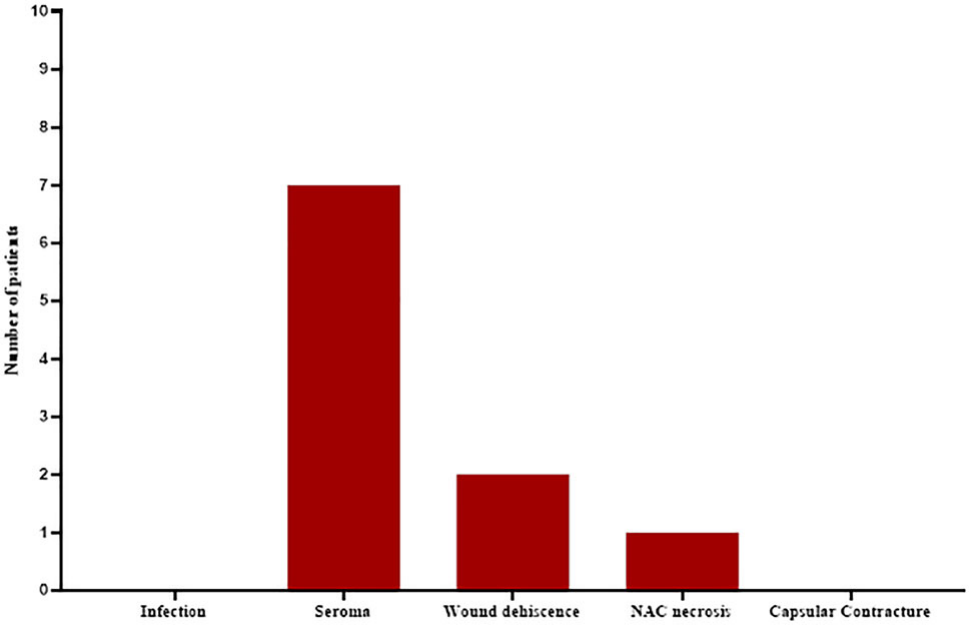
Bar chart shows the complications of laparoscopic camera sleeve for “no-touch” implant implant-based breast reconstruction

A total of 42 patients underwent IBBR using smooth round implants. The mean age of the patients was 46.5 ± 5.86 years. Of these, 34 (81%) patients underwent nipple-sparing mastectomy, and eight (19%) underwent skin-sparing mastectomy. The right breast was operated on in 18 patients (42.9%), while the left breast was operated on in 14 patients (33.3%). Ten patients (23.8%) underwent bilateral mastectomy. The mean implant size was 432.85 ± 69.68 ml. Postoperative complications included seroma in seven patients (16.7%) and wound dehiscence in two patients (4.8%). No cases of implant rupture, postoperative infection, or capsular contracture were observed during the follow-up period. Ten patients (23.8%) received adjuvant radiation therapy. The mean follow-up duration was 18.87 ± 8.86 months (Table 1 and Fig. 2).

**Table 1:** Baseline demographic characteristics and operative related data of the included patients

Variables	Number (%)/Mean ±SD
Age	46.5±5.86
Pattern of mastectomy	
Skin sparing mastectomy	8 (19%)
Nipple sparing mastectomy	34 (81%)
Laterality	
Right side	18 (42.9%)
Left side	14 (33.3%)
Bilateral	10 (23.8%)
Implant size	432.85±69.68
Complications	
Infection	0 (0%)
Seroma	7 (16.7%)
Wound dehiscence	2 (4.8%)
NAC necrosis	1 (2.4%)
Capsular contracture	0 (0%)
Postoperative radiotherapy	10 (23.8%)
Follow-up period (Months)	18.87±8.86

*SD* Standard deviation, *NAC* Nipple-areola complex

## Discussion

The terminal ducts of the breast are colonized with endogenous flora, which may contribute to contamination during breast implantation. Surgical preparation of the patient’s skin may not achieve complete sterility at the time of implant placement. Most infections occur in the immediate postoperative period, often due to inadequate skin preparation or suboptimal surgical technique. Several techniques have been employed to mitigate the risk of contamination, including the application of Tegaderm over the NAC, Ioban over the surgical field, irrigation with antibiotic solutions, and changing gloves prior to implantation. Despite these measures, the risk of infection and capsular contracture remains a significant factor affecting outcomes in implant-based breast surgery [23, 24]. This highlighted the need for a true no-touch technique, allowing breast implants to be placed directly into the pocket without contact with instruments, gloves, breast parenchyma, or the patient’s skin. The present study introduced a safe, simple, cost-effective, flexible, and disposable no-touch technique using a laparoscopic camera sleeve. Breast implantation with the sleeve was associated with no immediate postoperative infection or long-term capsular contracture. The sleeve allowed for the insertion of implants up to 545 ml, with a larger base opening accommodating larger implants, thereby reducing the risk of shell trauma and subsequent pain. The cut end of the sleeve was not sharp, minimizing the risk of implant damage, and no cases of rupture were observed. A new laparoscopic sleeve was used for each side, further reducing contamination risk compared with reusing a single sleeve or funnel.

The use of the laparoscopic camera sleeve for breast implantation was associated with no risk of infection and capsular contracture. This was consistent with Newman et al. 2018 who reported a lower risk of infection and capsular contracture after insertion of breast implants using the Keller funnel [16]. Moyer et al. [25] reported a 27-fold decrease in skin contact and parenchyma contamination; however, they reported an increase in contamination with increasing implant volume. Morkuzu et al. [15] review suggested that no-touch technique for breast augmentation or reconstruction is associated with shortened surgical time and incision length, along with lower risk of capsular contracture. Zhang et al. [26] proposed a Devon Lite Glove surgical light handle glove that can be easily converted into a sleeve by cutting off the end of the glove to fit in the IMF incision. However, this sleeve is only suitable for saline implants, highlighting the need for Keller Funnel to accommodate silicone implants [26]. This limited the further applicability of such a sleeve in the market owing to the rising trends in using silicone implants for breast augmentation and reconstruction [27]. Barker et al. [28] introduced the reversed glove sleeve technique as a no-touch technique, allowing insertion of breast implants up to 445 cm^3^ with no data regarding postoperative infection and capsular contracture.

The present study demonstrated a novel, effective, and reproducible no-touch technique for breast implantation. However, several factors may influence capsular contracture and implant-related complications that were not addressed. Furthermore, the exact timing for detecting capsular contracture remains uncertain, with most literature reporting 92% of cases within one year post-surgery [29]. There was no comparative arm to validate the outcomes of the laparoscopic camera sleeve to other no-touch techniques. Further randomized controlled trials with prolonged follow-up periods are needed to mitigate the current study’s limitations.

## Conclusions

The use of a laparoscopic camera sleeve represents a simple, cost-effective, and reproducible method for facilitating no-touch insertion of breast implants. This technique markedly minimizes skin contact and potential contamination of the breast parenchyma during IBBR, thereby decreasing the risk of postoperative infection and capsular contracture.

## Supplementary Information

Below is the link to the electronic supplementary material.

Supplementary file1 (MP4 79913 KB)

## Data Availability

The datasets used in the present study are available from the corresponding author on reasonable request.
